# P02-001 – A novel TNFRSF1A mutation in periodic fever

**DOI:** 10.1186/1546-0096-11-S1-A108

**Published:** 2013-11-08

**Authors:** E Erken, F Yıldız, D Arslan Taş, S Dinkci

**Affiliations:** 1 Rheumatology-Immunology, Çukurova University Faculty of Medicine, Adana, Turkey

## Introduction

Tumor necrosis factor receptor-associated periodic syndrome (TRAPS) is an autosomal dominant autoinflammatory disease characterized by periodic fever, accompanying with attacks of abdominal pain, arthralgia, myalgia, erythematous rashes, periorbital edema and conjunctivitis. Mutations in the extracellular domain of the 55-kD tumor necrosis factor receptor (TNFRSF1A) has been shown to be responsible for the TRAPS syndrome.

## Case report

A sixty two year old Turkish female presented with fever, periorbital edema, erythematous skin rash on the face, neck and arms at age 53 on her first admission. She had history of arthritic attacks on wrists, fingers, knees, elbows lasting 10-15 days, once or twice a year, which began at the age of twelve. Since the age of 50 she has experienced febrile attacks accompanying with abdominal pain, lasting 3-4 days in every 3-4 months. After the age of 53 she had attacks of pruritic erythematous rash on the neck, arms, legs and face, and bilateral periorbital edema as well lasting 10-14 days, once or twice a year. She had no family history of periodic fever. At age 55, she was diagnosed as primary Sjögren’s syndrome, with the findings of parotitis, dry eyes, positive Schirmer’s test and serum ANA and Anti-SSA positivity. Methyl prednisolon (MP) 8 mg/day and hydroxy chloroquine 400 mg/day was started. One year later, at age 56, her erythematous, pruritic skin lesions repeated with periorbital edema. MP was given 32 mg/day. ESR and CRP levels were elevated during attacks (eg. ESR:52 mm/h, CRP:17.4 g/L). Her skin lesions disappeared after commencement of MP. In attack free period, ESR and CRP returned to normal levels (eg. ESR:11 mm/h, CRP<5 g/L). MEFV mutation analysis of Exons 2 and 10 were negative by sequencing. For her periodic fever symptoms such as 39ºC fever accompanying with abdominal pain and skin lesions, TNFRS1 mutations were analyzed. Exon 2,3,4,5,6,7 and intron 2-3, 4-5 and 6-7 mutations were analyzed by polymerase chain reaction/sequence based typing technique. A novel mutation on exon 7 (S168C C>G, p.Ser197Cys) was identified.

## Discussion

This patient represents a novel mutation of TNFRSF1A in a Turkish patient with signs and symptoms of TRAPS syndrome, accompanying with primary Sjögren’s syndrome. As reported in Japanese cases previously, this patient with a novel mutation, has milder disease and her attacks of fever and rash respond well to glucocorticoid therapy. Coexistence of Sjögren’s syndrome may have masked the clinical manifestations of TRAPS in this patient. Without any family history, in this particular patient de novo TNFRSF1A mutation is possible.

## Disclosure of interest

None declared.
